# Inactivation Effect of Thymoquinone on *Alicyclobacillus acidoterrestris* Vegetative Cells, Spores, and Biofilms

**DOI:** 10.3389/fmicb.2021.679808

**Published:** 2021-06-02

**Authors:** Qiuxia Fan, Cheng Liu, Zhenpeng Gao, Zhongqiu Hu, Zhouli Wang, Jianbo Xiao, Yahong Yuan, Tianli Yue

**Affiliations:** ^1^College of Food Science and Engineering, Northwest A&F University, Yangling, China; ^2^Laboratory of Quality and Safety Risk Assessment for Agro-Products (Yangling), Ministry of Agriculture, Yangling, China; ^3^College of Food Science and Technology, Northwest University, Xi’an, China; ^4^Department of Analytical Chemistry and Food Science, Faculty of Food Science and Technology, University of Vigo-Ourense Campus, Ourense, Spain

**Keywords:** spore-forming bacteria, *Alicyclobacillus acidoterrestris*, *Nigella sativa*, thymoquinone, inactivation effect, spores, biofilm

## Abstract

*Alicyclobacillus acidoterrestris* (*A. acidoterrestris*), a spore-forming bacterium, has become a main challenge and concern for the juices and acid beverage industry across the world due to its thermo-acidophilic characteristic. Thymoquinone (TQ) is one of the active components derived from *Nigella sativa* seeds. The objective of this study was to investigate antibacterial activity and associated molecular mechanism of TQ against *A. acidoterrestris* vegetative cells, and to evaluate effects of TQ on *A. acidoterrestris* spores and biofilms formed on polystyrene and stainless steel surfaces. Minimum inhibitory concentrations of TQ against five tested *A. acidoterrestris* strains ranged from 32 to 64 μg/mL. TQ could destroy bacterial cell morphology and membrane integrity in a concentration-dependent manner. Field-emission scanning electron microscopy observation showed that TQ caused abnormal morphology of spores and thus exerted a killing effect on spores. Moreover, TQ was effective in inactivating and removing *A. acidoterrestris* mature biofilms. These findings indicated that TQ is promising as a new alternative to control *A. acidoterrestris* and thereby reduce associated contamination and deterioration in the juice and acid beverage industry.

## Introduction

*A. acidoterrestris*, a gram-positive, thermo-acidophilic, and spore-forming spoilage bacterium, is mainly responsible for the deterioration of acid products especially fruit juice, vegetable juice, and other relevant beverages (Keiichi and GOTO, 2006; Smit et al., 2011). This bacterium is extensively distributed in almost all components of the production and processing chain including the orchard, picking basket, fruit granary, fruit surface, cleaning water, pipeline water, production line, concentrated juice, semi-products, and final products (Chen et al., 2006; Driks et al., 2013; Huang et al., 2015). Compared to other food-associated bacteria, *A. acidoterrestris* has a greater tolerance to high temperatures and low pH environments. Moreover, under poor growth conditions, this microorganism can form endospores that are more resistant to thermo-acidic environments than vegetative cells and that could germinate, grow, and cause juice spoilage after reconstitution (Savaş Bahçeci et al., 2004). The characteristic of *A. acidoterrestris* grants it the ability to survive the commercial pasteurization process and also makes it a key quality control target for pasteurized fruit juices and relevant beverages. Juice deterioration by *A. acidoterrestris* resulted in the generation of guaiacol and halophenol compounds that were characterized by medicinal, smoky, and antiseptic tastes (Cai et al., 2015). The off-flavors of these compounds undermined the quality of the products (Pornpukdeewattana et al., 2020), which led to unaccepted products and substantial economic losses for the beverage industry.

Due to their distinctive structure, bacterial biofilms can form a barrier which will lessen and avoid the contact of bacterial cells in biofilms with disinfectants or antibacterial agents. Therefore, these biofilms cannot be inactivated or removed as easily as planktonic cells. Based on previous studies, *A. acidoterrestris* forms biofilms on different types of surfaces at food processing facilities including stainless steel, nylon, glass, and other surfaces (dos Anjos et al., 2013; Shemesh et al., 2014). It has been reported that *A. acidoterrestris* biofilm formation could be affected by many factors, such as strains, contact surface, and culture conditions (Basson et al., 2007; Prado et al., 2018). For the juice and beverage industry, the formation of *A. acidoterrestris* biofilms on the processing chain will lead to cross-contamination and thus increase the chance of product deterioration.

Currently, the food industry mainly relies on heat treatment, synthetic preservatives, and disinfectants to control contamination caused by *A. acidoterrestris*. Nevertheless, the former method affected the sensory quality of products, including color, flavor, and freshness, and ultimately caused reduced nutritional value. Meanwhile, the latter two methods (synthetic preservatives and disinfectants) may lead to adverse effects on human health. Consequently, there is a great need to search for safe, effective, and non-thermal methods to control *A. acidoterrestris* and its biofilms. As a non-thermal approach, natural compounds possessing antimicrobial activity has attracted more and more attention in recent years, especially phytochemicals.

*Nigella sativa* seeds (black seeds) have been used as a spice for thousands of years in the Middle East, India, and Pakistan. In Africa and Asia, these seeds were also used as a folk medicine to treat some diseases (Sutton et al., 2014). Thymoquinone (TQ, 2-methyl-5-isopropyl-1,4-benzoquinone), one of the functional constituents of the essential oil of *Nigella sativa* seeds, exhibits various pharmaceutical activities including anti-tumor, anti-inflammatory, anti-allergic, anti-diabetic, antimicrobial, and hepatoprotective effects (Chehl et al., 2009; Aziz et al., 2011; Forouzanfar et al., 2014; Abdelrazek et al., 2018; Noorbakhsh et al., 2018; Yang et al., 2015). In addition, TQ was reported to be able to inhibit biofilm formation by some pathogenic bacteria including *Cronobacter sakazakii* (Shi et al., 2017), *Pseudomonas aeruginosa* (Chakraborty et al., 2021), *Vibrio parahaemolyticus* (Guo et al., 2019), and *Listeria monocytogenes* (Liu et al., 2020). However, there are few studies that focus on the inactivation and removal effects of TQ on mature bacterial biofilms. Also, the effect of TQ on *A. acidoterrestris* vegetative cells, spores, and biofilms is very limited. In this study, the antimicrobial activity and possible mechanism of TQ against *A. acidoterrestris* vegetative cells were explored by MIC determinations, growth curve analysis, time-kill assay, SEM, and confocal laser scanning microscopy (CLSM) observations. Moreover, the effect of TQ on *A. acidoterrestris* spores was evaluated by viable counts and FESEM observation. And the inactivation and removal efficacy of TQ on *A. acidoterrestris* biofilms were assessed by viable cell counts and FESEM observation.

## Materials and Methods

### Reagents

TQ (HPLC ≥99%, CAS 490-91-5) was purchased from Aladdin Biochemical Technology Co., Ltd (Shanghai, China). The commercial compound was dissolved in dimethyl sulfoxide (DMSO) and diluted with sterile deionized water or *A. acidoterrestris* medium (AAM, 2.0 g of glucose, 2.0 g of yeast extract, 1.0 g of magnesium sulfate, 1.2 g of potassium dihydrogen phosphate, 0.2 g of ammonium sulfate, and 0.25 g of calcium chloride per liter of distilled water) before use. The final concentration of DMSO in all sample solutions was 1% (v/v). All other chemicals and reagents were of analytical grade.

### Bacterial Strains and Culture Conditions

The DSM 3922, DSM 3923, and DSM 2498 strains of *A. acidoterrestris* were purchased from the German Resource Centre for Biological Material (DSMZ). Two other *A. acidoterrestris* isolates (GYL-02, GYL-11) were obtained from our laboratory strain collection, and originated from the fruit granary of Shaanxi province. Minimum inhibitory concentrations (MICs) determination experiments were conducted for all five *A. acidoterrestris* strains; however, only DSM 3922 was used in subsequent assays. The strains were stored in AAM broth containing 25% (v/v) glycerol at −80°C before use. *A. acidoterrestris* working suspensions were prepared as follows: First, stock cultures were inoculated onto AAM plates and incubated at 45°C for 24 h. Then, the bacterial colony was transferred to AAM broth and incubated at 45°C for 12 h with shaking at 120 rpm. Next, the bacterial suspensions were centrifuged (4,000 × g, 5 min, 4°C), washed twice with phosphate buffered saline (PBS), and resuspended in sterile AAM broth. Then the optical density of the suspension at 600 nm (OD_600__ nm_) was adjusted to 0.5 for use.

To prepare spore suspension, the methods described by Cai et al. (2019b) and Prado et al. (2019) were followed with some modifications. *A. acidoterrestris* vegetative cells were cultured in AAM broth at 45°C for 15 days until about 90% sporulation was observed under a phase contrast microscopy. Subsequently, the cell culture was centrifuged (4,000 × g, 10 min, 4°C), washed twice, and resuspended in cold sterile saline. The spore concentration of the suspension was determined by heat shock (80°C for 10 min), serial dilution, and plating on AAM agar, which proved to be ∼10^6^ spores/mL. Finally, the suspension was stored at 4°C until use.

### MICs Determination

The MICs of TQ against five *A. acidoterrestris* strains were measured using a modified version of the broth dilution method, based on the guidelines of the Clinical and Laboratory Standards Institute (Clinical and Laboratory Standards Institute (CLSI), 2009). *A. acidoterrestris* suspensions (OD_600__ nm_ ≈ 0.5) were diluted in AAM broth to achieve the working suspension with a cell concentration of ∼10^5^ CFU/mL. Subsequently, the suspension was transferred to a 96-well plate, followed by the addition of TQ solutions. The final concentrations of TQ were 0 (control), 8, 16, 32, 64, 128, 256, and 512 μg/mL, respectively. AAM broth containing 1% DMSO but no TQ was taken as negative control. The plates were incubated at 45°C for 24 h, and the lowest concentration at which no visible bacterial growth was observed was considered as the MIC of TQ.

### Growth Curves

*A. acidoterrestris* DSM 3922 suspensions with an OD_600__ nm_ of 0.2 (∼3 × 10^4^ CFU/mL) were inoculated into the wells of a 96-well plate. TQ was added into the corresponding wells to obtain the final concentrations of 0 (control), 1/16 × MIC, 1/8 × MIC, 1/4 × MIC, 1/2 × MIC, and 1 × MIC, respectively. Then the plate was incubated at 45°C for 24 h and the OD_600__ nm_ values were measured by a multimode plate reader (Tecan, Infinite^TM^ M200 PRO, Mannedorf, Switzerland), at 2-h intervals in order to monitor the growth of *A. acidoterrestris* cells.

### Time-kill Assay

To assess the bactericidal activity of TQ against *A. acidoterrestris*, the time-kill assay was performed as described by Shi et al. (2016). *A. acidoterrestris* DSM 3922 suspensions were diluted in AAM broth to a cell concentration of ∼10^5^ CFU/mL. TQ was added to the suspensions to achieve final concentrations of 0 (control), 1/2 × MIC, 1 × MIC, and 2 × MIC, respectively. Then the samples were incubated at 45°C for 0, 3, 6, 9, 12, or 24 h. At each time point, samples were diluted in sterile PBS and spread onto AAM agar for viable enumeration.

### Field-Emission Scanning Electron Microscopy (FESEM) Observation

Following the method of Kang et al. (2019), FESEM observation was conducted with slight modifications. Briefly, *A. acidoterrestris* cells treated with TQ (0, 1 × MIC, and 2 × MIC) were incubated at 45°C for 2 h, followed by centrifugation (3,000 × g, 5 min, 4°C) and washing twice with PBS. The *A. acidoterrestris* cells were fixed with 2.5% glutaraldehyde at 4°C for 12 h and dehydrated with 30, 50, 70, 80, 90, and 100% water-ethanol solutions for 10 min, respectively. After critical point drying and gold spray treatment, samples were observed using a field-emission scanning electron microscope (Nano SEM-450, FEI, United States).

### Confocal Laser Scanning Microscopy (CLSM) Observation

CLSM observation was carried out according to the method described by Song et al. (2019), with minor modifications. *A. acidoterrestris* DSM 3922 suspensions treated with TQ (0, 1 × MIC, and 2 × MIC) were incubated at 45°C for 60 min. Then the suspensions were centrifuged (3,000 × g, 5 min, 4°C) and resuspended with 0.85% sterile saline. Following the addition of propidium iodide (PI) and SYTO 9, the suspensions were incubated in the dark for 15 min. Finally, the samples were deposited on a glass slide for CLSM analysis at the excitation/emission wavelengths of 480/500 nm (SYTO 9) and 490/635 nm (PI).

### Spore Inactivation Assay

With slight modifications, the method of Prado et al. (2019) was used in this assay. Briefly, *A. acidoterrestris* spore suspensions (stored at 4°C for about 1 month) were diluted with 0.85% sterile saline to achieve a cell density of 10^4^ ∼ 10^5^ CFU/mL. TQ was then added to obtain final concentrations of 0 (control), 4 × MIC, 8 × MIC, and 16 × MIC, respectively. The spores in the control group were not treated with TQ. Then samples were incubated at 45°C for 0, 30, 60, 120, 180, 240, or 360 min. At each time point, samples were taken, diluted, and spread onto AAM agar, followed by incubation and viable counts.

### FESEM Observation of Spores

To confirm the inactivation effect of TQ on spores, the morphology of TQ-treated spores was observed using FESEM. The spore suspensions mentioned above were treated with TQ (0, 4 × MIC, 8 × MIC, and 16 × MIC) at 45°C for 6 h. Samples were centrifuged at 4,000 × g at 4°C for 10 min and the cells were fixed with 2.5% glutaraldehyde. The following gradient dehydration, critical point drying, gold spray treatment, and FESEM observations were the same as described in above section.

### Biofilm Inactivation Assay

#### Inactivation of Biofilms Formed on a Polystyrene Microplate

According to the method by Fan et al. (2018), the effect of TQ on *A. acidoterrestris* biofilms formed on a polystyrene microplate was explored with some modifications. *A. acidoterrestris* DSM 3922 suspensions were inoculated into a 96-well flat-bottomed plate, and incubated at 45°C for 24 h to form biofilms. The AAM broth in wells were abandoned, and each well was gently rinsed with PBS. TQ solutions (prepared in AAM broth) at the concentrations of 0 (control), 2 × MIC, 4 × MIC, and 8 × MIC were added, and the plate was incubated at 45°C for 0, 30, 60, 90, 120, or 180 min. At each time point, the sample in each well was mixed thoroughly and then serially diluted in sterile PBS. One hundred microliters of the PBS suspension was spread onto AAM agar and the plates were incubated at 45°C for 24 h. The viable bacterial counts were expressed as log CFU/mL.

#### Inactivation of Biofilms Formed on a Stainless Steel Surface

Brifely, *A. acidoterrestris* DSM 3922 suspensions were added into sterile tubes with 1 mL per tube, followed by the addition of stainless steel sheets (1 cm × 1 cm) which had been cleaned by ultrasonication and sterilized with steam. Then samples were incubated at 45°C for 24 h. The sheets were gently rinsed with PBS and carefully transferred to new tubes containing TQ solutions of 0 (control), 2 × MIC, 4 × MIC, and 8 × MIC. After that, samples were incubated at 45°C for 0, 30, 60, 90, 120, or 180 min. At each time point, 1 g of sterile glass beads (1 mm, Glastechnique Mfg., Germany) was added into the tube and vortexed for 5 min (Kim et al., 2019; Huang et al., 2020). The resulting suspension was serially diluted with sterile PBS, followed by plating spread, incubation, and enumeration.

### Biofilm Removal Assay

With minor modifications, the method described by Fan et al. (2018) was followed. *A. acidoterrestris* DSM 3922 suspensions were co-incubated with sterile stainless steel sheets (5 mm × 5 mm) in a sterile 24-well flat-bottomed plate at 45°C for 24 h. The sheets were gently rinsed with PBS and carefully transferred to the new wells containing TQ solutions at 0 (control), 2 × MIC, 4 × MIC, and 8 × MIC. After an incubation at 45°C for 4 h, the sheets were washed gently with PBS before being immersed in a 2.5% glutaraldehyde solution for fixing. The following gradient dehydration, critical point drying, gold spray treatment, and FESEM analysis were conducted as described in the above section.

### Statistical Analyses

The IBM SPSS software (New York, United States) was used for statistical analysis. All experiments were performed three times, and the data were expressed as mean ± standard deviation. The differences between means were tested by two-way analysis of variance and were considered significant at *p* < 0.05.

## Results

### MICs

As shown in Table 1, the MICs of TQ against five *A. acidoterrestris* strains ranged from 32 to 64 μg/mL. The results suggested that TQ was effective at inhibiting *A. acidoterrestris* and may be used as a potential method to control *A. acidoterrestris* in the food industry.

**TABLE 1:** Minimum inhibitory concentrations (MICs) of TQ against *A. acidoterrestris* strains.

**Strains**	**Origins**	**MICs (μ g/ml)**
DSM 3922	DSM	32
DSM 3923	DSM	32
DSM 2498	DSM	32
GYL-02	Fruit granary	64
GYL-11	Fruit granary	32

**DSM, German Resource Centre for Biological Material.**

### Growth Curve Analyses

**FIGURE 1:**
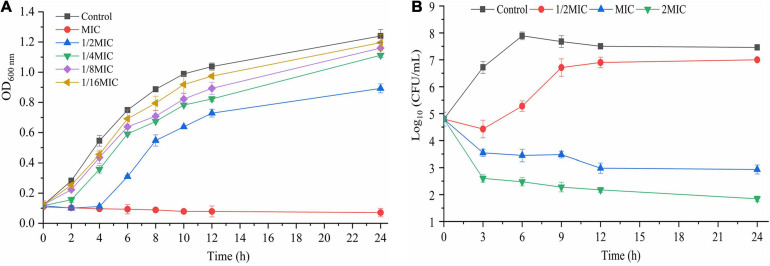
Growth curves **(A)** of *A. acidoterrestris* DSM 3922 in the presence of TQ (1 × MIC∼1/16 × MIC). Time-kill curves **(B)** of TQ (0, 1/2 × MIC, 1 × MIC, and 2 × MIC) against *A. acidoterrestris* DSM 3922 in AAM broth. Error bars represent standard deviation of three replicates. OD 600 nm, optical density at 600 nm.

The effect of TQ on the growth of *A. acidoterrestris* DSM 3922 is presented in Figure 1A. In the presence of TQ (1 × MIC), the OD_600__ nm_ of the *A. acidoterrestris* suspension remained steady within 24 h, suggesting that this concentration of TQ inhibited the growth of *A. acidoterrestris*. In the presence of TQ (1/4 × MIC, 1/8 × MIC, and 1/16 × MIC), the OD_600__ nm_ of the *A. acidoterrestris* suspension increased continuously over time, indicating that TQ at these concentrations could not inhibit the bacterial growth.

### Time-Kill Curves

The bactericidal effect of TQ on *A. acidoterrestris* DSM 3922 is shown in Figure 1B. The initial number of *A. acidoterrestris* cells in the samples was 4.8 log CFU/mL. Viable cells counts in the group treated with TQ (1/2 × MIC) decreased to 4.4 log CFU/mL and then increased continually to 7.1 log CFU/mL. In contrast, the number of cells in groups exposed to TQ (1 × MIC and 2 × MIC) reduced constantly over time. At 3 h post-treatment, TQ at 1 × MIC and 2 × MIC reduced the cell counts to 3.5 and 2.6 log CFU/mL, respectively. And at 24 h post-treatment, TQ at the two concentrations caused greater reductions to 2.9 and 1.8 log CFU/mL, respectively. The results revealed that TQ at 1 × MIC and 2 × MIC exerted a bactericidal effect on *A. acidoterrestris* cells.

### FESEM Observation

**FIGURE 2:**
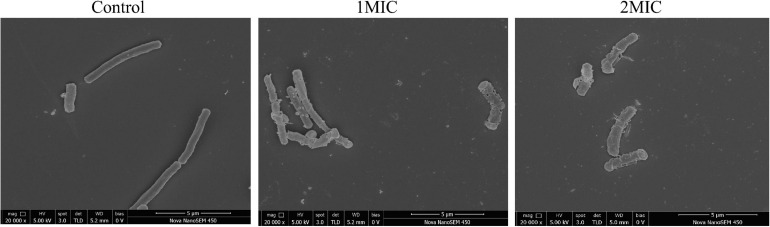
Scanning electronic images of *A. acidoterrestris* DSM 3922 after exposure to TQ of 0 (control), 1 × MIC and 2 × MIC for 2 h.

The changes in *A. acidoterrestris* cell morphology caused by TQ were confirmed by FESEM observation. As shown in Figure 2, untreated *A. acidoterrestris* cells were regular, smooth, plump, and rod-shaped. Whereas after exposure to TQ (1 × MIC and 2 × MIC) for 2 h, the surfaces of the *A. acidoterrestris* cells became rough, irregular, and wrinkled. Moreover, perforations and cankers were observed. The results revealed that TQ at 1 × MIC and 2 × MIC destroyed the cell morphology of *A. acidoterrestris*.

### CLSM Observation

**FIGURE 3:**
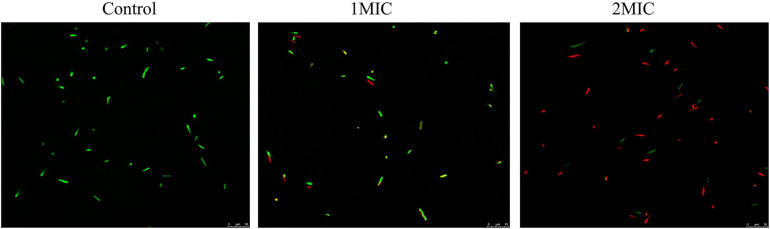
Confocal laser scanning microscopy images of *A. acidoterrestris* DSM 3922 after treatment with TQ of 0 (control), 1 × MIC and 2 × MIC for 60 min. Scale bar: 10 μm.

The damage of TQ to *A. acidoterrestris* membrane integrity was assessed using fluorescent dyes SYTO 9 and PI by CLSM observation. SYTO 9 is a nucleic acid dye which can penetrate all bacterial cells, combine with DNA, and generate green fluorescence. By contrast, PI only stains the bacterial cells that have a damaged cell membrane and it generates red fluorescence. As presented in Figure 3, *A. acidoterrestris* cells in the control group exhibited complete green fluorescence and almost no red fluorescence was observed, suggesting that cells in this group had an intact membrane. In contrast, a small amount of red fluorescence occurred after treatment with TQ at 1 × MIC, indicating that the membrane integrity of some cells was destroyed by TQ. After exposure to TQ at 2 × MIC, a dramatic increase in red fluorescence intensity was observed, showing that this concentration of TQ resulted in a more damaged cell membrane. Overall, these findings demonstrated that TQ could impair the cell membrane of *A. acidoterrestris* in a dose-dependent manner.

### Spore Inactivation

**FIGURE 4:**
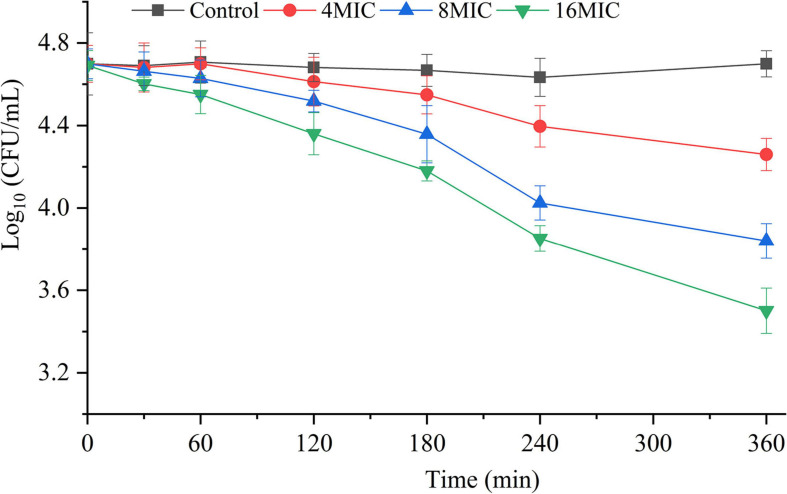
Inactivation effect of TQ on *A. acidoterrestris* spores. The spores were treated with TQ at the concentrations of 0 (control), 4 × MIC, 8 × MIC, and 16 × MIC for 360 min. Error bars represent standard deviation of three replicates.

The killing effect of TQ (0, 4 × MIC, 8 × MIC, and 16 × MIC) on *A. acidoterrestris* spores is shown in Figure 4. The number of spores at 0 min in all samples were 4.7 log CFU/mL. Obviously, the number of spores in the untreated group remained the same throughout the experiment, while the number of spores in the TQ-treated groups decreased over time. At 240 min post-treatment, TQ (4 × MIC, 8 × MIC, and 16 × MIC) reduced the spore counts to 4.4, 4.0, and 3.8 log CFU/mL, respectively. And greater reductions to 4.2, 3.8, and 3.5 log CFU/mL were achieved at 360 min post-treatment. The results revealed that TQ exerted a moderate inactivation effect on *A. acidoterrestris* spores.

### FESEM Observation of Spores

**FIGURE 5:**
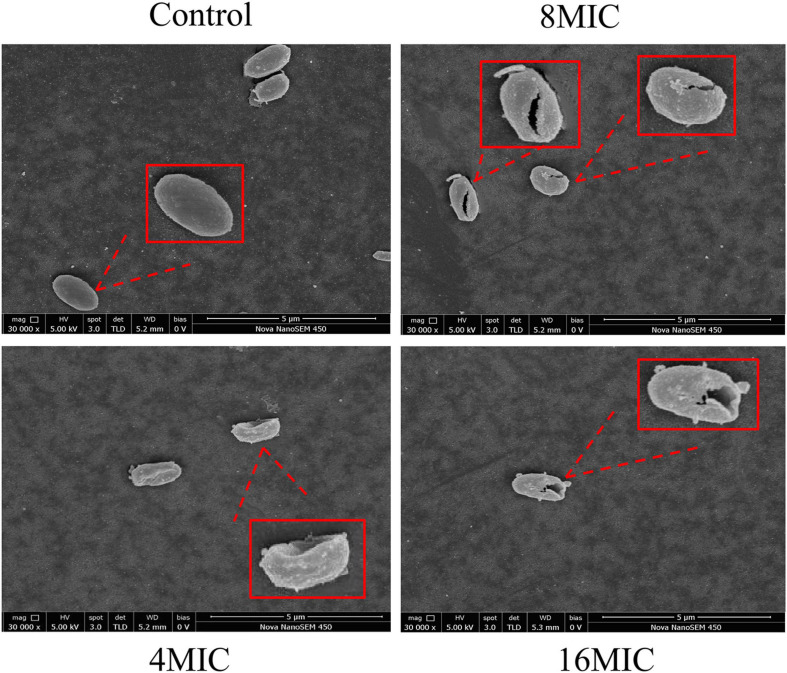
Scanning electronic images of *A. acidoterrestris* DSM 3922 spores after treatment with TQ of 0 (control), 4 × MIC, 8 × MIC, and 16 × MIC for 6 h.

The effect of TQ on *A. acidoterrestris* spores was further verified by FESEM observation. As presented in Figure 5, untreated spores exhibited a normal morphology with an oval, smooth, and plump shape. After exposure to TQ (4 × MIC) for 6 h, the surface of spores became rough, wrinkled, or even hollow. Moreover, rupturing and cracks occurred on the surface as the concentration of TQ increased to 8 × MIC and 16 × MIC.

### Biofilm Inactivation

**FIGURE 6:**
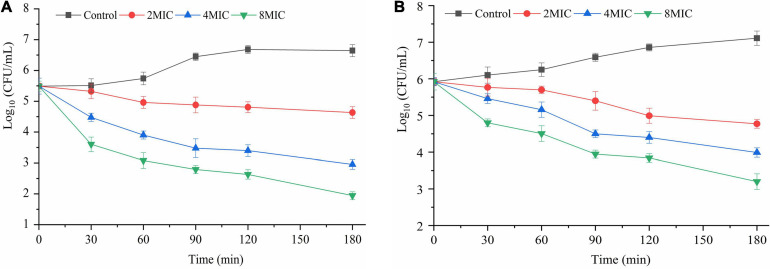
Inactivation effect of TQ on *A. acidoterrestris* DSM 3922 biofilms grown in AAM broth at 45°C for 24 h on polystyrene **(A)** and stainless steel **(B)** surfaces. Formed biofilms were treated with TQ at the concentrations of 0 (control), 2 × MIC, 4 × MIC, and 8 × MIC for 180 min. Error bars represent standard deviation of three replicates.

The inactivation effects of TQ on *A. acidoterrestris* biofilms formed on polystyrene and stainless steel surfaces are shown in Figures 6A,B. Obviously, TQ treatments reduced the number of viable cells in biofilms formed on the two surfaces. The initial number of *A. acidoterrestris* cells on biofilms formed on the polystyrene surface was 5.5 log CFU/ml and decreased to 4.9, 3.8, 3.0 log CFU/ml, 4.8, 3.4, 2.6 log CFU/ml, and 4.6, 2.9, 1.9 log CFU/ml after treatment with TQ (2 × MIC, 4 × MIC, 8 × MIC) for 60, 120, and 180 min, respectively. Similarly, TQ reduced the number of *A. acidoterrestris* cells in biofilms formed on the stainless steel surface. At the initiation of treatment, the total number of viable *A. acidoterrestris* cells on stainless steel surfaces was 5.9 log CFU/ml. Whereas at 60, 120, and 180 min post-treatment of TQ (2 × MIC, 4 × MIC, 8 × MIC), viable counts in the biofilms were reduced to 5.6, 5.1, 4.5 log CFU/ml, 5.0, 4.4, 3.8 log CFU/ml, and 4.7, 3.9, 3.2 log CFU/ml, respectively. The results indicated that TQ could inactivate *A. acidoterrestris* cells on biofilms developed on polystyrene and stainless steel surfaces in a concentration-dependent manner.

### Biofilm Removal

**FIGURE 7:**
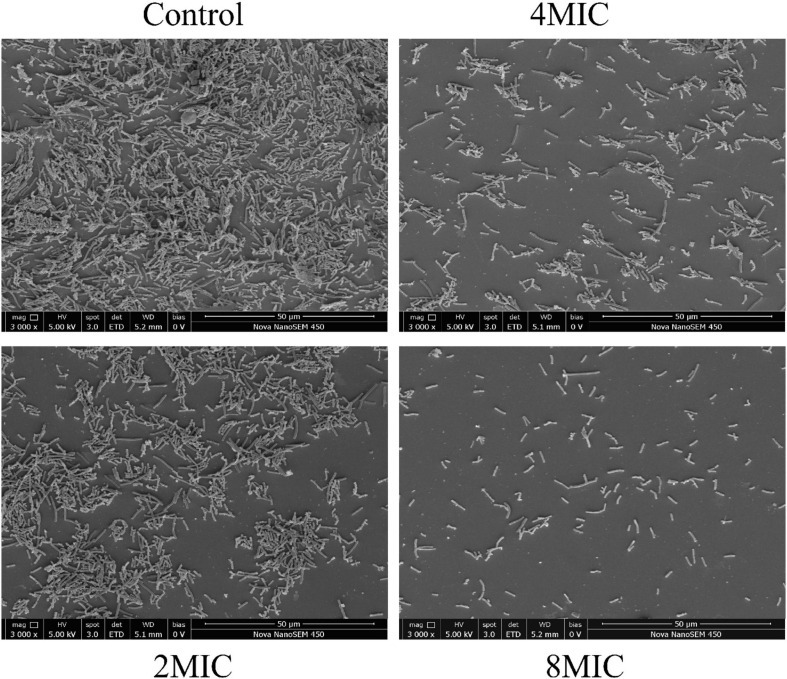
Scanning electronic images of *A. acidoterrestris* DSM 3922 biofilms after exposure to TQ of 0 (control), 2 × MIC, 4 × MIC, and 8 × MIC for 4 h.

The elimination effect of TQ on *A. acidoterrestris* biofilms was confirmed by FESEM observation and the images are presented in Figure 7. *A. acidoterrestris* biofilms in the control group were dense, evenly distributed, and had a multilayer structure with clustered cells. Nevertheless, at 4 h post-treatment of TQ (2 × MIC and 4 × MIC), the layer became thin, uneven, and the cell clusters in the biofilm decreased. After treatment with TQ at 8 × MIC, the number of *A. acidoterrestris* cells adhered to the surface decreased significantly and no *A. acidoterrestris* clusters were observed. The results suggested that TQ may be applied as an antibiofilm agent in the food industry to control *A. acidoterrestris* biofilms.

## Discussion

In this study, TQ was demonstrated to possess good antimicrobial activity against *A. acidoterrestris* vegetative cells with MICs ranging from 32 to 64 μg/mL. In recent years, natural compounds with antibacterial activity have garnered great attention as an efficient means of fighting bacteria. Many natural compounds has been reported to show an inhibitory effect on *A. acidoterrestris*, such as paracin C, pomegranate fruit extract, rosemary extracts, and lemon essential oil (Cristina Maldonado et al., 2013; Molva and Baysal, 2015; Piskernik et al., 2016; Pei et al., 2017). Also, previous studies demonstrated that *Piper marginatum* extracts, thymol, cinnamic acid, and chlorogenic acid had the ability to inhibit growth of *A. acidoterrestris* with MICs of 62.5, 250, 375, and 2,000 μg/mL (de Pascoli et al., 2018; Cai et al., 2019a,b). Obviously, TQ showed a stronger inhibitory activity against *A. acidoterrestris* than most of the reported compounds. From growth curves, we can easily see that the cell density of samples treated with TQ at 1 × MIC was almost unchanged over time (Figure 1A), suggesting that the treatment completely suppressed the growth of *A. acidoterrestris* DSM 3922 cells. Similarly, the time-kill curves in Figure 1B showed that the number of bacterial cells decreased significantly after exposure to TQ at 1 × MIC. Therefore, TQ (1 × MIC) also has a bactericidal effect on *A. acidoterrestris* DSM 3922.

Normal cell morphology and an intact cell membrane are essential for bacteria to stay alive and maintain various physiological metabolism activities. At present, natural products from different sources are a research hotspot in the antibacterial field, and have great potential as novel bacteriostats and bactericides. Many natural compounds were demonstrated to exert antibacterial activity by destroying cell morphology and membrane integrity. Xu et al. (2017) investigated the effect of punicalagin on *Staphylococcus aureus* morphology using scanning and transmission electron microscopy, and found that abnormal cell morphology (the enlarged size and rough surface) occurred after the treatment of punicalagin at 0.5 mg/mL. Sun et al. (2020) proved that chlorogenic acid induced the cell envelope damage of *Salmonella Enteritidis* by scanning electron microscopy observation. And further CLSM examination demonstrated that the inner membrane was also disrupted by chlorogenic acid. In this study, TQ at the concentration of MIC and 2MIC (32 and 64 μg/mL) induced abnormal morphology of *A. acidoterrestris*, destroyed the integrity of the cell membrane, and thus resulted in growth inhibition and cell death (Figures 2, 3).

*A. acidoterrestris* is a spore-former, and the strong resistance of its spores to high temperature and other adverse environments, such as low pH conditions, has make the bacterium a challenge in the quality control of pasteurized juices and beverages (Jovetta et al., 2011). Therefore, many strategies have been explored by scholars to control *A. acidoterrestris* spores, including chemical and physical methods. In the present study, TQ was proved to inactivate spores at the concentrations of 4 × MIC, 8 × MIC, and 16 × MIC (128, 256, and 512 μg/mL) (Figure 4). Moreover, TQ treatment altered the morphology of spores and rendered their surfaces rough, hollow, and cracked (Figure 5). Similarly, Cai et al. (2019b) demonstrated that thymol was able to inhibit and inactivate *A. acidoterrestris* spores, with an MIC and MBC of 0.5 mg/mL and greater than 1.0 mg/mL, respectively. Prado et al. (2019) reported that *A. acidoterrestris* spores counts were reduced by 3 log CFU/mL after exposure to ultraviolet C radiation from 5 to 15 min. Besides, like TQ, ultraviolet C radiation (12.6 kJ/m^2^) produced visible morphological changes in *A. acidoterrestris* spores such as distortions, central depression, and expressive roughness. In addition to single chemical and physical methods, combinations of these methods were also used to control *A. acidoterrestris* spores. For example, Huertas et al. (2014) demonstrated that the inactivation effects of nisin and citral on *A. acidoterrestris* spores were enhanced by heat. Similarly, high pressure processing pretreatment promoted the inactivation of *A. acidoterrestris* spores by thermo-sonication (Evelyn and Silva, 2016). Based on these findings, we proposed to study the synergistic spore-inactivation effects of combinations of TQ with different physical methods, such as ozone, ultrasound, thermal treatment, ultraviolet radiation, and high pressure treatments.

*A. acidoterrestris* was able to form biofilms on the surfaces of food processing facilities, such as stainless steel, glass, and nylon (dos Anjos et al., 2013; Shemesh et al., 2014). Traditionally, chemical disinfectants have been the primary method to control bacterial biofilms. Nevertheless, the safety and residue of these sanitizers went against people’s health concepts and become increasingly unaccepted (Fan et al., 2018). On the contrary, natural compounds possessing antibiofilm activity are attracting a growing amount of attention. According to previous studies, some natural compounds can inhibit the formation of these biofilms. For example, clove essential oil (0.05%) can reduce the formation of *A. acidoterrestris* biofilms on glass and polyvinyl chloride surfaces by 25.1∼65.0% (Kunicka-Styczyńska et al., 2020). Consistently, ultraviolet C radiation was proven to exhibit an inhibitory effect on *A. acidoterrestris* biofilms formation on stainless steel and rubber surfaces, and also an inactivation effect on a biofilm formed on two surfaces (Prado et al., 2019). Besides, an earlier study proved the bactericidal efficacy of three sanitizers (peracetic acid, sodium hypochlorite, and quaternary ammonia) on *A. acidoterrestris* biofilms formed on stainless steel, polyvinyl chloride, and nylon surfaces (dos Anjos et al., 2013). However, literature concerning inactivation and elimination of *A. acidoterrestris* biofilms by natural compounds is very limited. In the current study, TQ, at 2 × MIC, 4 × MIC, and 8 × MIC, was demonstrated to be effective in inactivating and removing *A. acidoterrestris* biofilms on polystyrene and stainless steel surfaces, and resulted in reductions of 2.0∼4.7 and 2.5∼3.6 log CFU/mL after 180-min treatment, compared with the control group (Figures 6, 7). These results suggested that TQ can be used as a natural agent to control and eliminate *A. acidoterrestris* biofilms formed on polystyrene plastic and stainless steel surfaces in the food production line. Further studies are required to explore other natural compounds that possess antibiofilm activity against *A. acidoterrestris*.

## Conclusion

In conclusion, TQ showed good antibacterial activity against *A. acidoterrestris* vegetative cells with MICs of 32∼64 μg/mL. TQ exerted its antimicrobial and bactericidal activity by delaying bacterial growth, altering cell morphology (shrinkage, perforation, and canker), and destroying the cytoplasmic membrane. In addition, TQ showed a moderate killing effect on *A. acidoterrestris* spores and was effective in inactivating *A. acidoterrestris* biofilms formed on polystyrene and stainless steel surfaces as well as in removing *A. acidoterrestris* biofilms. These findings demonstrate that TQ has the potential to be an effective antimicrobial and antibiofilm agent to control contamination and deterioration caused by *A. acidoterrestris*.

## Data Availability Statement

The original contributions presented in the study are included in the article/supplementary material, further inquiries can be directed to the corresponding author/s.

## Author Contributions

QF, YY, and TY conceived and designed the experiments. QF, CL, and ZW performed the experiments. ZG and ZH analyzed the data. JX contributed to reagents and materials. QF wrote the manuscript. YY and TY provided funding acquisition. All authors read and approved the manuscript.

## Conflict of Interest

The authors declare that the research was conducted in the absence of any commercial or financial relationships that could be construed as a potential conflict of interest.
